# The Anrep effect mediates myocardial contractility and stroke work in a patient with severe pulmonary valve stenosis before and after successful valvuloplasty: a case report

**DOI:** 10.1093/ehjcr/ytag627

**Published:** 2026-08-26

**Authors:** Jan-Christian Reil, Gert-Hinrich Reil, Volker Rudolph, Werner Scholtz

**Affiliations:** Ruhr-Universität Bochum, Herz- und Diabeteszentrum NRW, Klinik für Allgemeine und Interventionelle Kardiologie/Angiologie, Georgstrasse 11, 32547 Bad Oeynhausen, Deutschland; Universitätsklinik für Innere Medizin—Kardiologie, Klinikum Oldenburg, Rahel Straus- Strasse 10, 26131 Oldenburg, Germany; Ruhr-Universität Bochum, Herz- und Diabeteszentrum NRW, Klinik für Allgemeine und Interventionelle Kardiologie/Angiologie, Georgstrasse 11, 32547 Bad Oeynhausen, Deutschland; Ruhr-Universität Bochum, Herz- und Diabeteszentrum NRW, Klinik für Allgemeine und Interventionelle Kardiologie/Angiologie, Georgstrasse 11, 32547 Bad Oeynhausen, Deutschland

**Keywords:** Anrep effect, Contractility, Pulmonary stenosis, Right ventricle, Pressure–volume (PV) analysis, Case report

## Abstract

**Background:**

Acute Anrep effect describes transient afterload-dependent increase in myocardial contractility with the aim of maintaining a stroke volume during physical exertion. Sustained activation of the Anrep effect has recently been identified as a compensatory mechanism in afterload-driven diseases, such as hypertrophic obstructive cardiomyopathy or aortic stenosis. The aim of this case presentation was to show whether the Anrep effect can also be chronically activated and reversed in the right heart in a patient with pulmonary stenosis.

**Case summary:**

A 30-year-old female patient with chronic exertional dyspnoea (New York heart association II) and suspected supravalvular pulmonary stenosis was referred to our hospital for further diagnosis and treatment. Echocardiography revealed a borderline hypertrophied right ventricle with a gradient of more than 100 mmHg across the pulmonary valve. However, it was not possible to distinguish between valvular and supravalvular stenosis. Pulmonary stenosis with characteristic bulging of the valve could finally be diagnosed by transoesophageal echocardiography. Subsequently, a successful balloon valvuloplasty of the pulmonary valve was performed, which significantly reduced the maximum pressure gradient from 70 to 19 mmHg. Detailed pressure–volume analyses demonstrated distinctive increased contractility (end-systolic elastance = 1.47 mmHg/mL), increased afterload effective arterial elastance, prolonged systole duration (480 ms), and increased stroke work prior to valvuloplasty consistent with the Anrep effect. After successful intervention, the Anrep effect abruptly reversed characterized by reduction of afterload, contractility, and systolic duration.

**Conclusion:**

This case report of severe pulmonary stenosis identified a possible contribution of the chronic Anrep effect as a regulator of right ventricular contractility and energy expenditure before and after successful pulmonary valve implantation.

Learning points

- Non-invasive pressure–volume analysis can reveal pathophysiological relationships that go beyond the purely descriptive, morphological standard parameters of echocardiography.
- The Anrep effect likely also regulates right ventricular function in patients with chronically elevated afterload like severe pulmonary valve stenosis.

## Introduction

The Anrep effect causes a rapid increase in myocardial contractility in response to a sudden increase in afterload at high energetic costs.^1^ This regulatory mechanism enables the heart to maintain stroke volume (SV) and cardiac output despite increased peripheral resistance and is independent of preload, heart rate, and catecholamines.^1,2^ The Anrep effect shows a characteristic haemodynamic triad of increased afterload, increased contractility, and prolonged systolic duration.^3,4^

While the Anrep effect is traditionally regarded as a short-term physiological adaptation to physical exertion, even in the right heart,^5^ recent studies have shown that it can also occur as a chronic mechanism in diseases with persistently high afterload, such as severe aortic valve stenosis^6^ or hypertrophic obstructive cardiomyopathy.^3^ It is currently unclear whether the Anrep effect also occurs chronically in the right ventricle (RV), although some findings in patients with pulmonary arterial hypertension support this idea.^7^ We therefore examined the case of a 30-year-old patient with severe pulmonary valve (PV) stenosis, paying particular attention to the Anrep effect before and after successful valvuloplasty.

## Summary figure

**Figure:**
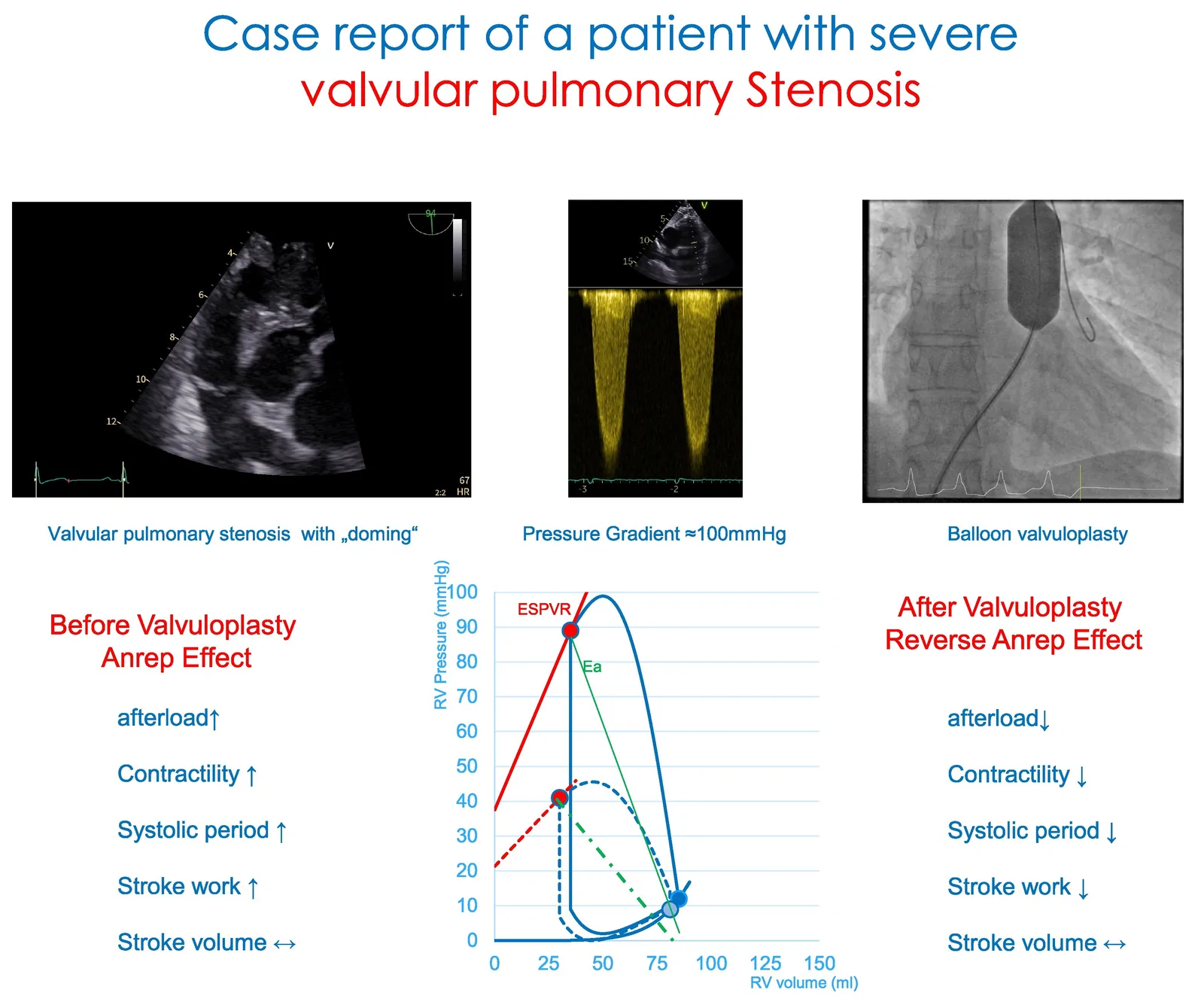


## Case presentation

**Figure 1:**
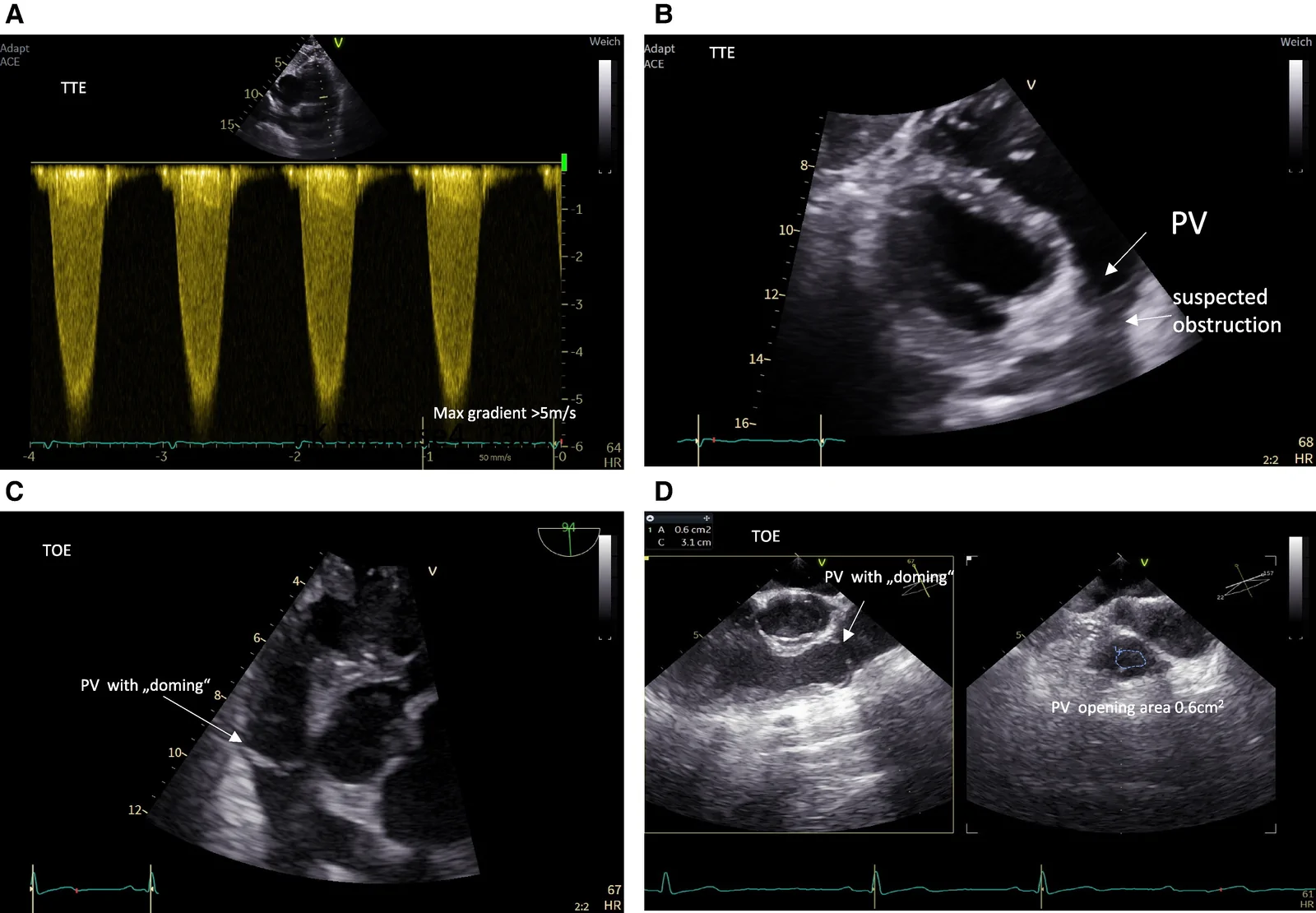
(*A*) Doppler gradient with maximum velocity of more than 5 m/s across the pulmonary valve was recorded. (*B*) Transthoracic echocardiographic image of pulmonary valve stenosis, unable to differentiate between supravalvular and valvular stenosis. (*C* and *D*) Initial transoesophageal echocardiography images clearly show valvular stenosis with doming, which mimics supravalvular stenosis, with a valve opening area of 0.6 cm^2^.

A 30-year-old slim woman was referred to our hospital with suspected severe supravalvular PV (pulmonary valve) stenosis diagnosed by an external magnetic resonance imaging. The patient complained of exertional dyspnoea (New York heart association II) and had no other medical conditions. Auscultation revealed a systolic murmur of 4/6 with maximum intensity above the second intercostal space left parasternal. Transthoracic echocardiography documented a significantly increased maximum gradient of >100 mmHg (*Figure 1A*) across the valve with a normally wide but borderline hypertrophied RV (RV wall thickness 5 mm), but was unable to clearly localize the stenosis in the RV outflow tract. Transoesophageal echocardiography (TEE) finally clearly identified ‘valvular’ pulmonary stenosis with fused valve leaflets protruding from their attachment into the pulmonary artery (PA) as a conical, windsock-like structure (opening area 0.6 cm^2^), mimicking a supposed supravalvular stenosis (*Figure 1C* and *D*).

**Figure 2:**
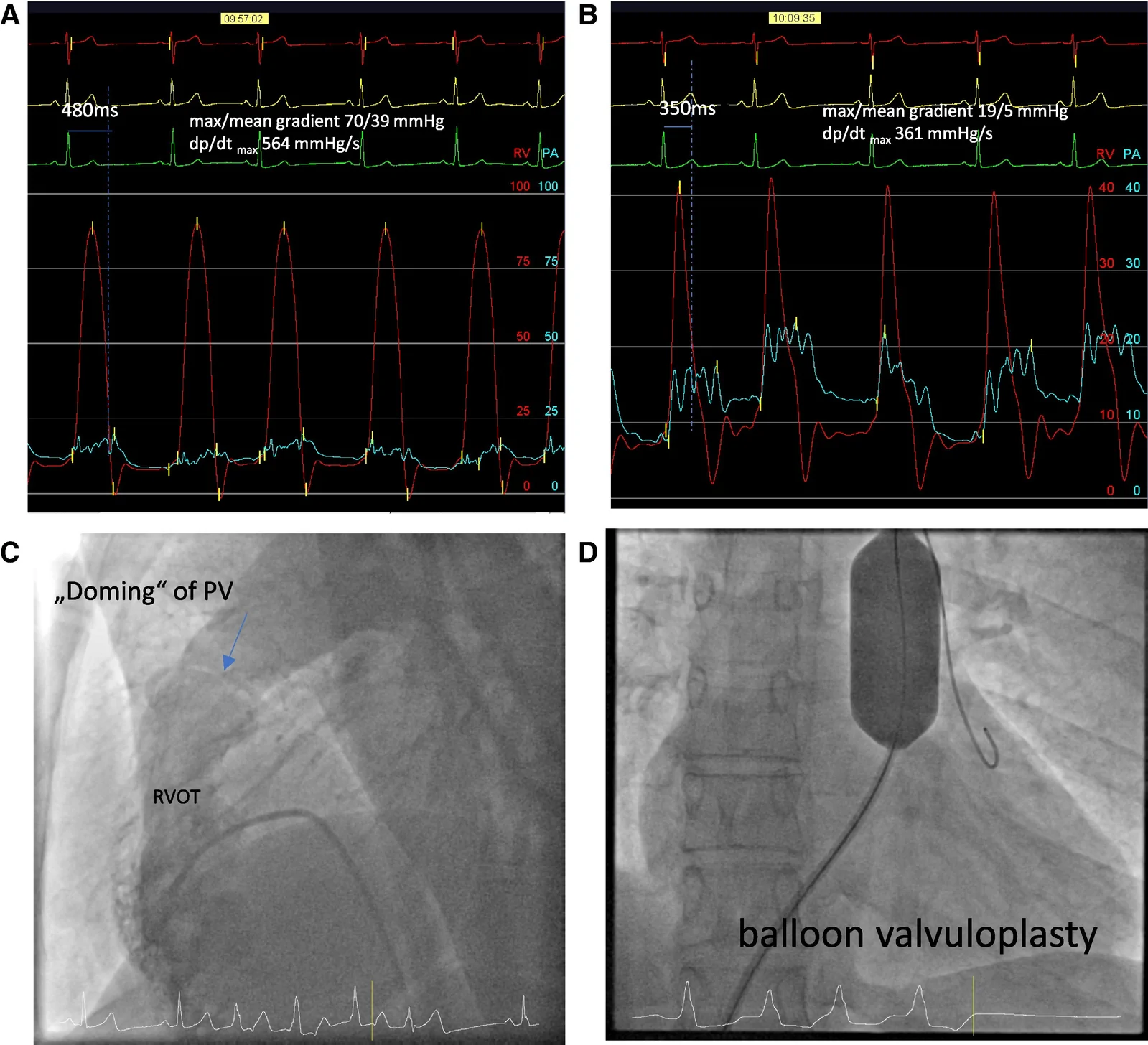
(*A*) Original recording with right ventricular pressure curve (red) and pulmonary artery pressure curve (blue): scale **0–100 mmHg**. Systolic period including isovolumetric contraction and ejection phase is measured between R-wave of the ECG and the intersection of the descending right ventricular pressure curve with the pulmonary artery pressure curve (pulmonary valve closure = 480 ms). Together with increased afterload (right ventricular end-systolic pressure 90 mmHg) and contractility (dp/dt_max_ 564 mmHg/s), the Anrep triad can thus be demonstrated clinically. (*B*) Original recording with right ventricular pressure curve (red) and pulmonary artery pressure curve (blue): scale **0–40 mmHg**. Systolic period is measured between R-wave of ECG and the intersection of the descending right ventricular pressure curve with the pulmonary artery pressure curve (pulmonary valve closure = 350 ms). Together with lower afterload (right ventricular end-systolic pressure 41 mmHg) and contractility (dp/dt_max_ 361 mmHg/s), the reversed Anrep triad can thus be demonstrated clinically. (*C*) Angiographic image of the pulmonary valve with doming. (*D*) Angiographic image of valvuloplasty.

The treatment of choice for PV stenosis is balloon valvuloplasty, which was performed under conscious sedation with a 22 mm balloon on our patient without complications (*Figure 2*). The gradient dropped acutely from max/mean gradient of 70/39 mmHg to 19/5 mmHg. Pressure–volume loops were reconstructed from invasive pressure measurements (*Figure 2*) and echo-guided 3D volume sets obtained before and immediately after the procedure to obtain more precise haemodynamic insights. Contractility parameters, such as end-systolic pressure–volume relationship (ESPVR) with its slope end-systolic elastance (Ees), were calculated from a correlation according to Richter.^8^ The area of the individual pressure–volume loop reflects the stroke work (SW), while the area under the ESPVR to the left of the pressure–volume loop reflects the potential work (PE). The sum of SW and PE corresponds to the total area in the pressure–volume diagram [pressure–volume area (PVA)] and represents the total energy consumed, which correlates with oxygen demand.^9^ The effective arterial elastance (Ea) represents the total afterload of the RV, calculated from the ratio of right ventricular end-systolic pressure (RVESP) to SV.^10^

**Figure 3:**
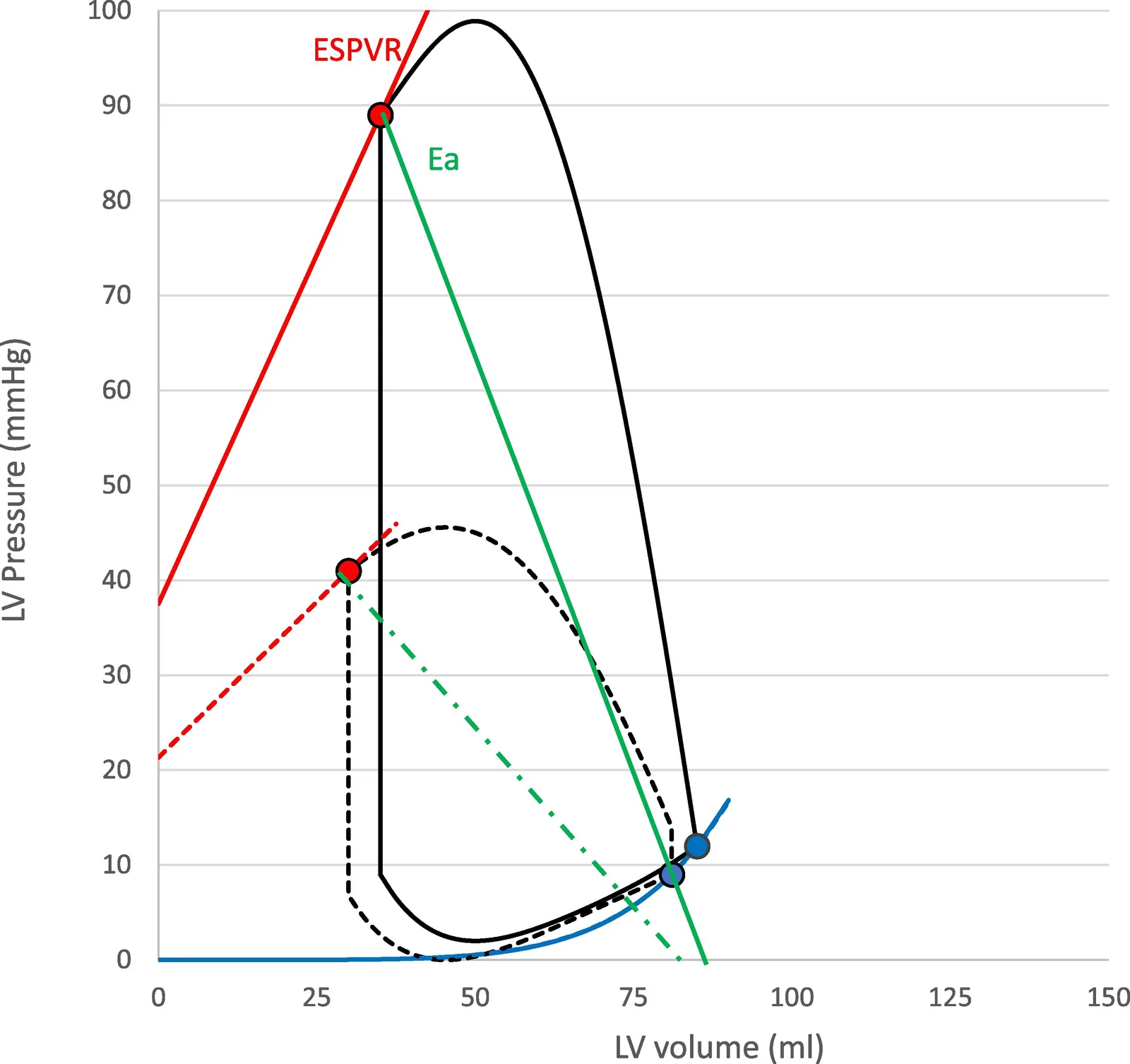
Representative pressure–volume loops before (solid lines) and after (dashed lines) pulmonary valve valvuloplasty. Red ESPVR, end-systolic pressure–volume relationship; green Ea, effective arterial elastance.

Before valvuloplasty, afterload was elevated (steeper slope of Ea, green solid line) with a concomitant significant increase in contractility (ESPVR with steeper slope Ees, red solid line) and prolonged systolic duration (*Figure 3*; *Table 1*, 480 ms). This triad of parameters is suggestive for the Anrep effect and resulted in an increase in total energy per beat (PVA) in order to maintain SV. After valvuloplasty, the Anrep effect was reversed with a reduction in afterload (flattening of Ea, green dotted line) and contractility (flattening of ESPVR, red dotted line) and a shortening of systolic duration (*Figure 3*, 350 ms), so that an almost identical SV could be achieved compared with the pre-intervention values, but with less SW.

**Table 1:** Hemodynamic parameters

	Pre-PV-valvuloplasty	Post-PV-valvuloplasty	Change in parameters
Afterload			
Peak PV gradient (mmHg)	70	19	↓
RVESP (mmHg)	89	41	↓
Ea (mmHg/mL)	1.78	0.80	↓
Heart rate (beats/min)	50	54	↔
Wall stress (mmHg)^11^	101	51	↓
Energy			
SW (mmHg·mL)	1892	1155	↓
PE (mmHg·mL)	2433	1163	↓
PVA (mmHg·mL)	4326	2319	↓
Systolic function			
RVEF (%)	59	61	↔
SV (mL)	50	51	↔
dp/dt_max_ (mmHg/s)	564	361	↓
Ees (mmHg/mL)	1.47	0.66	↓
Vo (mL)	−25.5	−32.5	↓
Ea/Ees	1.21	1.14	↔
Systolic duration			
Systolic period (ms)	480	350	↓
Diastolic function			
EDP (mmHg)	12	10	↓
EDV (mL)	85	81	↓

Ea, effective arterial elastance; EDP, end-diastolic pressure; EDV, end-diastolic volume; Ees, end-systolic elastance; PE, potential energy; PV gradient, peak pulmonary valve gradient; PVA, pressure–volume area; RVEF, right ventricular ejection fraction; RVESP, right ventricular end-systolic pressure; SV, stroke volume; SW, stroke work; Vo, intersection of end-systolic pressure–volume relationship with volume axis (not shown).

## Discussion

Two findings appear to be important in this case. First, an accurate diagnosis of the subtype of pulmonary stenosis is important, as a distinction must be made between supra-, infra-, and valvular stenosis, which are treated differently. Second, the haemodynamic measurements strongly suggest chronic activation of the von Anrep effect in severe pulmonary stenosis and its resolution following successful valve implantation.

Pulmonary stenosis accounts for 10% of all congenital heart defects.^12^ The form characterized by fusion of the individual cusps of the valve with ‘doming’ is by far the most common subtype in 90% of cases and is shown in our patient.^13^ The treatment of choice is balloon valvuloplasty, which has been used since the early 1980s and has an excellent long-term prognosis.^12,14^ Other forms of pulmonary stenosis are treated purely surgically, which once again underscores the importance of correct diagnosis prior to intervention.

It is noteworthy that this case also points to the important but little-known presence of the Anrep effect in the RV. Direct evidence of the Anrep effect in the RV has so far only been demonstrated after acute PA clamping in patients undergoing lung resection.^5^ Similar to hypertrophic obstructive cardiomyopathy and aortic valve stenosis in the left ventricle,^3,6^ the Anrep effect can possibly be also chronically activated in the right heart. Some data on patients with pulmonary arterial hypertension also provide initial evidence of this idea.^7^ We therefore examined the case of a 30-year-old patient with severe PV stenosis, paying particular attention to the chronic Anrep effect before and after successful valvuloplasty.

The patient presented here demonstrates that, in the presence of high outflow resistance (PV stenosis), the chronic Anrep effect can maintain a constant SV of the RV by increasing contractility, although this accompanied by in high stroke work. Increased afterload and increased contractility, together with prolonged systolic duration, form the characteristic triad used to diagnose the Anrep effect in ventricles facing high afterload. This triad is indicative of the Anrep effect, as other haemodynamic determinants, such as positive inotropic effects by catecholamines or the Frank–Starling law, deviate from this typical constellation.^1^ A catecholamine-induced reduction in contractility after the procedure would be accompanied by a prolongation of systole—exactly the opposite of our results. Furthermore, the involvement of the Frank–Starling mechanism and the Bowditch effect in the altered haemodynamics following valve implantation can be ruled out, as the preload parameters (right ventricular end-diastolic pressure, end-diastolic volume) and heart rate remain virtually unchanged after the procedure (*Table 1*). Therefore, valvuloplasty with significant reduction in afterload induces the reverse Anrep effect with a decrease in the aforementioned parameters.

The case also highlights the importance of the pressure–volume diagram for revealing and interpreting complex and multifaceted haemodynamic mechanisms, as indicated by the close interaction between afterload, contractility, and SW. None of these findings can be detected using a standard echocardiogram. However, diagnosing the Anrep effect is relatively simple in clinical practice. Its significant triad can be easily detected using right heart catheter pressure measurements with electrocardiogram recording, as shown in *Figure 2A* and *B* (see legend).

## Conclusion

This case demonstrates the successful treatment of severe PV stenosis using valvuloplasty, which was originally classified as supravalvular stenosis due to ‘doming’ of the valve. Ventricular mechanics, particularly the contractility of the right heart, are likely controlled largely by the Anrep effect in this disease.

## Data Availability

All necessary data are included in the text, the table, or the figures.

## Lead author biography

Jan-Christian Reil completed his doctoral thesis at the Institute of Physiology at Ludwig-Maximilians-University, Munich, Germany, in 2005 and received his clinical training at the Department of Internal Medicine III/Cardiology in Homburg/Saar, Germany. From 2015 to 2022, he was head of the echo laboratory at the Lübeck Heart Centre, Universitätsklinikum Schleswig-Holstein, Campus Lübeck, Germany. From 2022 onwards, he has been the head of echo laboratory of the Heart and Diabetes Center (HDZ-NRW) in Bad Oeynhausen, Germany.

## Notes

**Consent:** The author(s) confirm that, in accordance with the COPE guidelines, the patient’s written consent has been obtained for the submission and publication of this case report, including the figures and accompanying text.
